# The Canadian Health Clock and health calculators

**DOI:** 10.17269/s41997-020-00348-9

**Published:** 2020-07-14

**Authors:** Bernard C. K. Choi, Douglas G. Manuel

**Affiliations:** 1 Centre for Surveillance and Applied Research, Public Health Agency of Canada, Government of Canada, Ottawa, Ontario Canada; 2 Dalla Lana School of Public Health, University of Toronto, Toronto, Canada; 3 Ottawa Hospital Research Institute, Ottawa, Ontario Canada; 4 Department of Family Medicine, and the School of Epidemiology and Public Health, University of Ottawa, Ottawa, Ontario Canada

**Keywords:** Health clock, Health calculator, Innovation, Data visualization, Information dissemination, Public health, Horloge de la santé, Calculateur de la santé, Innovation, Visualisation des données, Diffusion d’information, Santé publique

## Abstract

**Setting:**

This paper documents a participatory process of Health Portfolio staff in the design of a clock, and announces the 2020 Canadian Health Clock, with links to numerous online health calculators. The clock is part of the Health Portfolio’s celebration activities in 2019 of “100 Years of Health”, as the Department of Health was established in Canada in 1919.

**Intervention:**

The intervention was the development of a clock on the Government of Canada website with linkage to calculators as a health promotion tool. The clock was built on the concept of the 2004 Chronic Disease Clock, which shows the number of deaths so far today, and so far this year. The clock was developed using a consultative approach, following a review of the original clock.

**Outcomes:**

The 2020 clock incorporates new data visualization concepts. New features, facilitated by improved technology, include: expansion to all causes of death; blinking red dots to enhance visual impact; and three clock versions (analogue, featuring a moving circle; digital, table format; and graphical, bar chart format). The clock also provides links to a number of health calculators, to allow people to seek personalized information to improve their health.

**Implications:**

The online health clock and health calculators are good examples of innovation in health risk communication tools for effective knowledge translation and dissemination. They inform people about health statistics (clock) and their health (calculators). The clock engages people in the context of the Canadian population, whereas the calculators provide personalized information about improving an individual’s future health.

## Résumé

**Contexte:**

Ce document présente un processus participatif pour le personnel du portefeuille de la Santé relativement à la conception d’une horloge. Il annonce la création de l’horloge canadienne de la santé 2020, qui comprend des liens vers de nombreux calculateurs de la santé en ligne. L’horloge fait partie des activités de célébration de 2019 du portefeuille de la Santé pour souligner les « 100 ans d’expertise en santé », la création du ministère de la Santé au Canada remontant à 1919.

**Intervention:**

L’intervention consistait à créer une horloge sur le site Web du gouvernement du Canada et à y ajouter des liens vers des calculateurs comme outil de promotion de la santé. L’horloge a été conçue sur le modèle de l’horloge des maladies chroniques de 2004, qui montre le nombre de décès depuis le début de la journée et depuis le début de l’année. L’horloge a été élaborée selon une approche consultative, à la suite de l’examen de l’horloge originale.

**Résultats:**

L’horloge 2020 intègre de nouveaux concepts en matière de visualisation des données. Les nouvelles fonctionnalités, dont l’intégration a été simplifiée grâce à une technologie améliorée, comprennent notamment les suivantes : inclusion de toutes les causes de décès; points rouges clignotants pour accentuer l’effet visuel; et trois versions d’horloge (analogique, avec un cercle en mouvement; numérique, en format de tableau; et graphique, en format de diagramme). L’horloge contient aussi des liens vers un certain nombre de calculatrices de la santé afin de permettre aux personnes de trouver des renseignements personnalisés pour améliorer leur santé.

**Conséquences:**

L’horloge de la santé en ligne et les calculateurs de la santé sont de bons exemples d’outils novateurs de communication des risques pour la santé qui permettent une application et une diffusion efficaces des connaissances. Ces outils donnent aux gens la possibilité de se renseigner à la fois sur les statistiques en santé (horloge) et sur leur propre santé (calculateurs). L’horloge suscite l’intérêt des gens dans le contexte de la population canadienne, alors que les calculateurs offrent des renseignements personnalisés sur l’amélioration de la santé future d’une personne.

## Introduction

This paper, with the release of the 2020 Canadian Health Clock and a description of various online health calculators for life expectancy and health risk estimation, is part of the year-long Health Portfolio celebration activities in Canada in 2019 of “100 Years of Health”. The Health Portfolio comprises Health Canada, the Public Health Agency of Canada, the Canadian Institutes of Health Research, the Canadian Food Inspection Agency and the Patented Medicine Prices Review Board. On June 6, 1919, the Department of Health was created by the Department of Health Act (Parliament of Canada 1919).

The 2019 Health Portfolio activities celebrate achievements in health to date, and encourage innovations into the future. One celebration activity was to involve Health Portfolio staff in a hands-on intervention project to design an innovative health promotion tool for implementation on the Government of Canada website.

## Prologue

By looking back at the progress in health in the past 100 years, the health clock and multivariable health calculators, presented in this paper, are examples of how Canadian health data and the Internet are evolving the way disease and mortality statistics are reported. The clock raises awareness on the urgency and impact of diseases and injuries on population health, and incorporates new techniques of interactive and animated visualizations. The life expectancy and disease risk calculators raise awareness of preventable risks on personal or individual health, and provide personalized mortality and disease risk estimation.

The objectives of the Canadian Health Clock are as follows: first, to have a hands-on activity as part of celebration activities in 2019 of “100 Years of Health”; second, to raise awareness on the impact and urgency of disease burden on health of Canadians; and third, to motivate people to do more positive things about their health, using personalized feedback information from the online health risk calculators. The objective of the calculators is to inform people, based on their individual risk factor and exposure profile, how social and behavioural risks relate to their future health.

## The Canadian Health Clock

The Canadian Health Clock is a data visualization tool to disseminate public health information in real time. Instead of the usual annual number of deaths by cause of death, it shows the number of deaths so far today (as of 12:00 midnight) and so far this year (as of January 1). The numbers are ticking with a blinking red dot, and increasing in actual time like a clock.

**Fig. 1:**
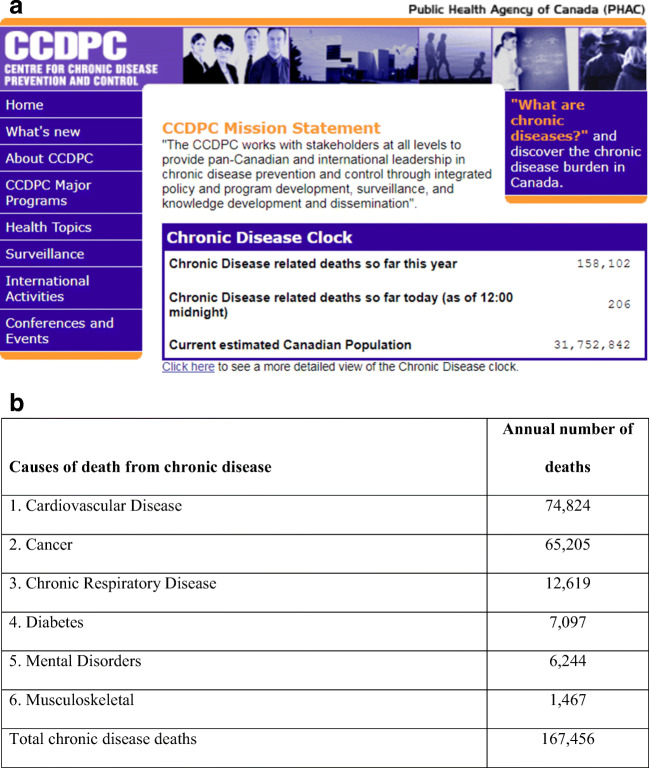
(a) The 2004 Chronic Disease Clock (Canada): A virtual clock set up on the Public Health Agency of Canada (PHAC)’s Centre for Chronic Disease Prevention and Control (CCDPC) website from 2004 to 2012 (now available on archive https://web.archive.org/web/20050407215825/http://www.phac-aspc.gc.ca:80/ccdpc-cpcmc/index_e.html). (b) Technical specifications for the 2004 Chronic Disease Clock (Canada). The 2004 Chronic Disease Clock is based on the most recently available mortality data in Canada which is 2001. Data are from Statistics Canada Health Statistics Division’s Canadian mortality data, obtained through Public Health Agency of Canada’s ORIUS database. Data are for Canada for both sexes and all ages combined. Chronic diseases, for the purpose of the Chronic Disease Clock, include the following six disease categories: cardiovascular disease (Circulatory Disease ICD-10* I00–I99), cancer (Neoplasm ICD-10 C00–D48), chronic respiratory disease (Respiratory Disease ICD-10 J00–J98 minus pneumonia ICD-10 J12–J18, minus influenza ICD-10 J10–J11 and minus bronchitis ICD-10 J40–J42), diabetes (ICD-10 E10–E14), mental disorders (ICD-10 F00–F89) and musculoskeletal (ICD-10 M00–M99). *ICD-10, International Classification of Diseases, 10th revision (WHO 2016)

**Fig. 2:**
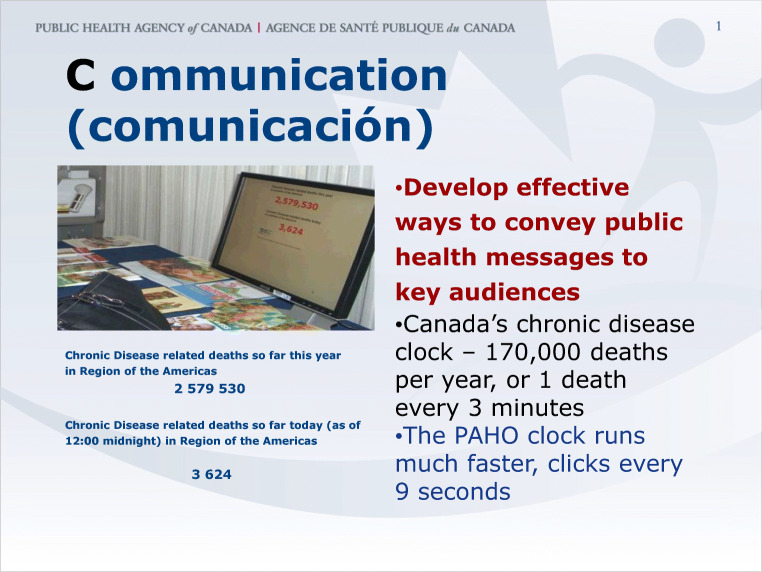
The 2007 Chronic Disease Clock (Region of the Americas): A physical clock set up on a laptop computer, powered by programs on a compact disc (CD), and displayed at the Pan American Health Organization (PAHO) Headquarters in Washington, DC, for World Heart Day on September 30, 2007

**Fig. 3:**
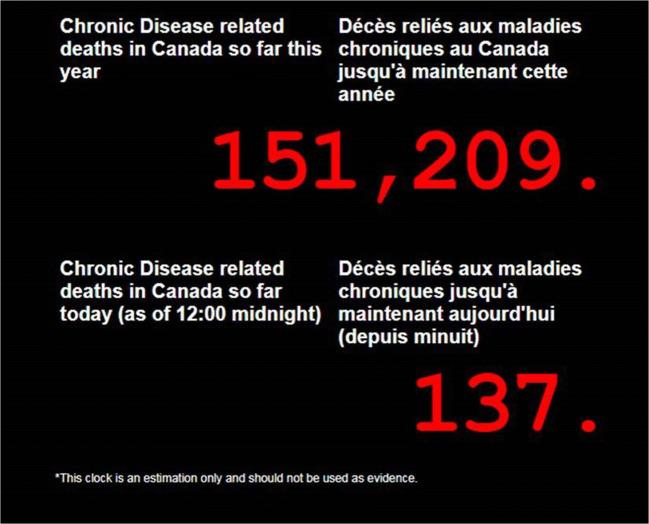
The 2008 Chronic Disease Clock: A physical clock set up on a laptop computer and announced by the Public Health Agency of Canada at the Chronic Disease Prevention Alliance of Canada (CDPAC) 2008 Conference in Ottawa, November 24–26, 2008

The 2020 Canadian Health Clock is based on the concept of the Chronic Disease Clock released by the Public Health Agency of Canada (PHAC) in 2004 (Fig. 1a) (Choi 2005a). To convert statistics to a form which people can visualize, the 2004 Chronic Disease Clock was a digital clock with deaths so far this year and so far today. People could actually watch the number of chronic disease deaths increase with time, because one such death occurred every 3 min in Canada (Choi 2005b). Technical specifications for the 2004 Chronic Disease Clock (Fig. 1b) show how the clock works. The 2004 clock featured an extended version which shows deaths in six categories: cardiovascular disease, cancer, chronic respiratory disease, diabetes, mental disorders and musculoskeletal diseases. Following the Canadian clock in 2004, a similar US Chronic Disease Clock was set up by the US Centers for Disease Control and Prevention (CDC) on its website in 2005. In 2007, the Pan American Health Organization (PAHO) invited PHAC to build a Chronic Disease Clock for the Region of the Americas (35 countries) for the occasion of World Heart Day. It was a real physical clock that included a laptop computer and a flat screen TV, and was placed in the lobby of the PAHO Headquarters in Washington, DC (Fig. 2). The Regional Clock increases the number of deaths once every 9 s. In 2008, in another version of the Chronic Disease Clock, a real clock on a laptop computer was set up and announced by PHAC at the Chronic Disease Prevention Alliance of Canada (CDPAC) 2008 Conference in Ottawa (Fig. 3).

**Fig. 4:**
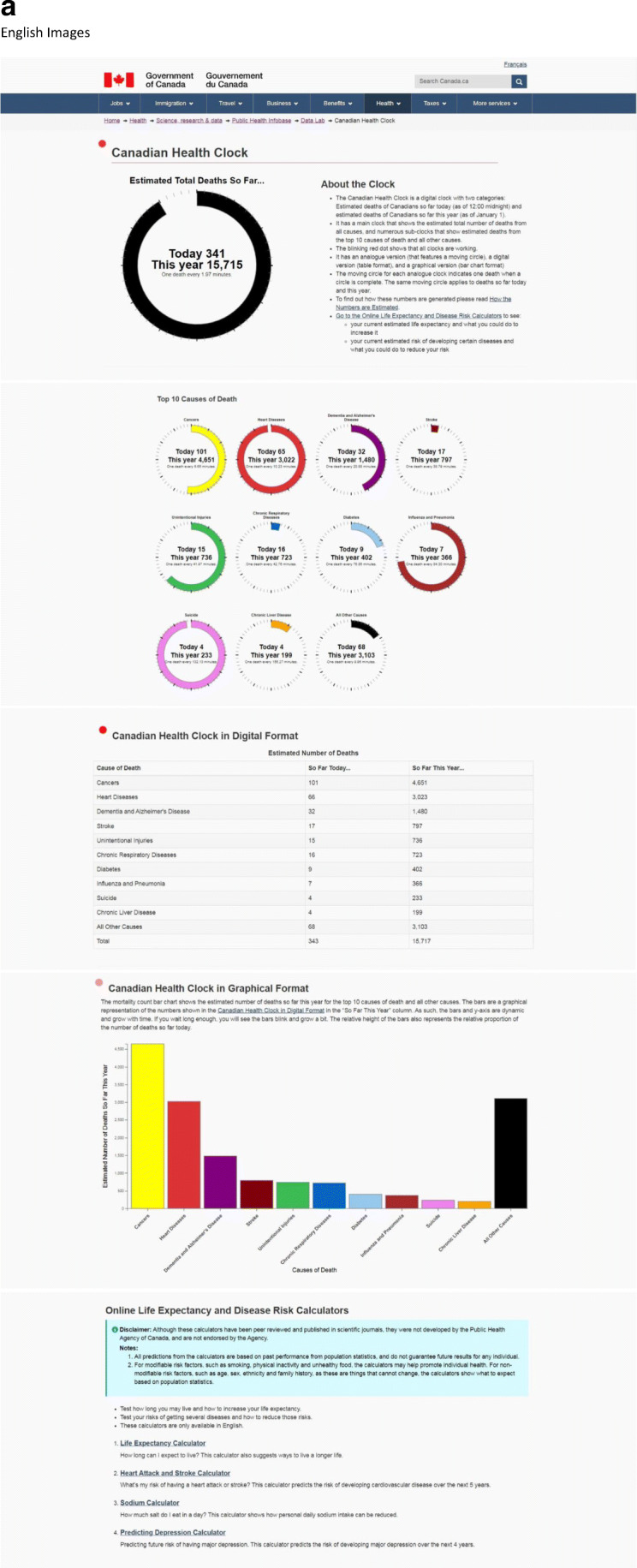

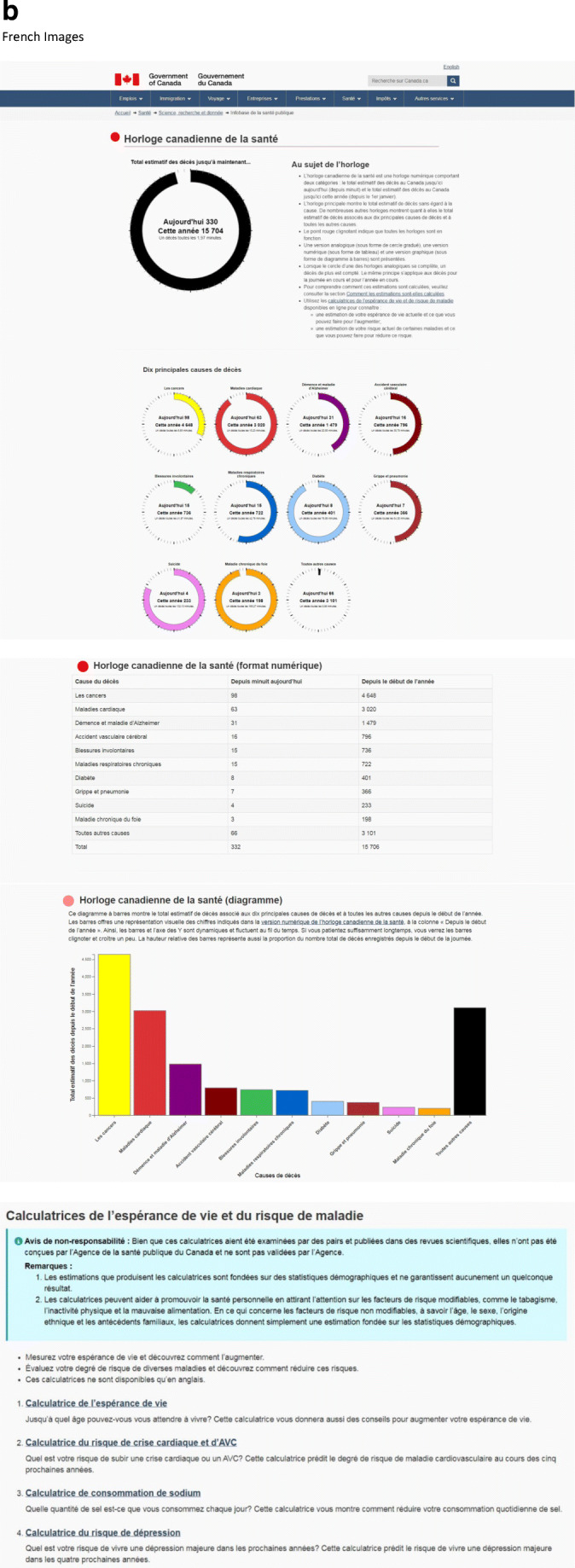
(**a**) The 2020 Canadian Health Clock: An online clock that shows the number of deaths so far today and so far this year, available on the Public Health Agency of Canada website, as part of the year-long celebration activities in Canada in 2019 of “100 Years of Health” ([English] https://health-infobase.canada.ca/datalab/canadian-health-clock.html [French] https://sante-infobase.canada.ca/labo-de-donnees/horloge-canadienne-de-la-sante.html). (**b**) Technical specifications for the 2020 Canadian Health Clock. The clock provides estimates that are based on retrospective data, that is, the most recent available mortality data. For example, the 2020 clock is based on actual 2016 mortality data (Fig. 4b). The numbers of deaths so far today (as of 12:00 midnight) and so far this year (as of January 1) are estimated by apportioning the annual number of deaths according to a year of 365.2422 days, and a day of 24.0000 h. Note that: (1) For simplicity, the same apportioning method applies to all different causes of death. For example, no special treatment is made for “Influenza and Pneumonia” to obtain daily death estimates excluding seasonal fluctuations. Therefore, during influenza seasons, the daily number of deaths from “Influenza and Pneumonia” is expected to be higher than that shown in the clock. (2) The estimates for deaths from chronic conditions are likely underestimates because many chronic conditions are often not a direct cause of death. For example, people with chronic obstructive pulmonary disease (COPD) often die of pneumonia. (3) The estimates for diabetes deaths are likely underestimates, given the limitations of death certificates to understanding diabetes deaths. (4) Deaths per day or per minute is popular, but it must be recognized that there is not an assembly line of death, and deaths are sporadic. (5) All estimated numbers of deaths so far today and this year, and estimated number of minutes to achieve one death, are based on apportioning (the assumption of an assembly line), and therefore should not be taken to suggest any level of precision that is not present. Annual numbers of deaths, by cause of death in ICD-10 codes*, for Canada (2016), are shown in Fig. 4b. Data sources for Fig. 4b: Data for all deaths and nine of the top 10 causes of death are obtained directly from publicly available online data from Statistics Canada for Canada for 2016, Table: 13-10-0394-01, which lists the annual numbers of deaths from the top 10 and top 50 causes of death in Canada from 2012 to 2016 (Reference: Statistics Canada, 2018. Table: 13-10-0394-01 (formerly CANSIM 102–0561). Leading causes of death, total population, by age group https://www150.statcan.gc.ca/t1/tbl1/en/tv.action?pid=1310039401). The number of deaths for “Dementia and Alzheimer’s Disease” is obtained by the Public Health Agency of Canada from custom tabulation from Statistics Canada. It is necessary to add dementia to Alzheimer’s disease as Statistics Canada uses a list developed by the US National Center for Health Statistics (NCHS) for ranking. Since dementia is not in the US list, it does not make it into Statistics Canada’s top 10 or even top 50 causes of death, even with 18,649 deaths, while Alzheimer’s disease (ICD-10 G30), in the US list, ranks number 7 with 6521 deaths in 2016. Together, dementia and Alzheimer’s disease ranks number 3. *ICD-10, International Classification of Diseases, 10th revision (WHO 2016)

The 2020 Canadian Health Clock (Fig. 4a) is available on the Government of Canada website:

- [English] https://health-infobase.canada.ca/datalab/canadian-health-clock.html
- [French] https://sante-infobase.canada.ca/labo-de-donnees/horloge-canadienne-de-la-sante.html

Technical specifications for the 2020 clock (Fig. 4b) show how this clock works.

As part of the celebration activities, during the 8 months from November 2018 to June 2019, more than a hundred Health Portfolio staff participated in the design, data source selection, naming and construction of the clock.

The 2020 Canadian Health Clock has several new features. First, it includes all causes of death, and not just chronic diseases. Second, it has a main clock that shows the total number of deaths from all causes, and numerous sub-clocks that show deaths from the top 10 causes of death and all other causes. Third, blinking red dots show that all the clocks are working and convey a sense of urgency. Fourth, it has an analogue version (that features a moving circle), a digital version (table format) and a graphical version (bar chart format) (Fig. 4a). Fifth, the moving circle for each analogue clock indicates one death when a circle is complete. The moving circles increase the visual impact and urgency conveyed by the clocks. Sixth, the bars and *y*-axis of the bar chart clock are dynamic and grow with time. If people wait long enough, they can see the bars blink, indicating one death, and grow a bit. Seventh, besides disseminating disease burden (negative health information), disease prevention and health promotion measures (positive health information) are now added to the clock to encourage healthy living among Canadians. This is done through links to a series of life expectancy and health risk calculators (Fig. 4a) (see next section).

The online clock encourages adaptation for use in other countries and continents. The computer source code (JavaScript) that powers the clock is open source, and is available via GitHub. Instructions are available on the clock website. The basic design of the clock is of a general nature which includes a “main clock” and a number of “sub-clocks”. The main clock can be “all deaths” (as in our released version) and the sub-clocks can be various categories of major causes of death, based on International Classification of Disease (ICD) (10th revision (WHO 2016)). Alternatively, the main clock can be “chronic disease deaths in Latin America” and the sub-clocks can be such deaths in various Latin American countries wanting to compare with one another. Additionally, the clock can be based on number of disease cases instead of number of deaths. It can be adapted for attributable deaths (e.g., smoking-related deaths, opioid-related deaths) and avoidable deaths. Use can be extended to risk factor clocks, healthy behaviour clocks, protective factor clocks and health inequity clocks (e.g., deaths by socio-economic status or geographic locations). Possibilities are many.

## The life expectancy and health risk calculators

Multivariable risk tools have been developed using clinical data and they are commonly used in the clinical setting. Canada has one of the largest ongoing population health surveys, the Canadian Community Health Survey (CCHS), and these surveys are among the few that are internationally linked to mortality and disease outcomes (Sanmartin et al. 2016). Uniquely, these data allow for the development of multivariable risk tools using nationally represented surveillance data that can translate socio-demographic and health behaviour risks into personalized information (Manuel et al. 2012, 2016).

Life expectancy’s appeal is its intuitive meaning for both population and individual uses. Of life expectancy, Nathan Keyfitz, one of the world’s most prominent demographers who started his career at Statistics Canada, stated: “Like other successful models, the life table has given shape to the natural world; we are incapable of thinking of population change and mortality from any other starting point” (Keyfitz 1977).

It could be difficult for people to remember the annual deaths or the death rate per 100,000 person-years, or to visualize what these numbers mean to them. However, they probably would understand better if they are told Canada’s life expectancy at birth (males 79.9 years, females 84.0 years) (Statistics Canada n.d.) and also that Canada has one of the highest ranking life expectancies worldwide. Life expectancy is calculated using no other information than mortality rates, but the data are translated into a meaningful life course perspective. As individuals, people do not have an annual mortality rate (a measure only for populations) but they each have an annual mortality risk between 0 and 1. Their mortality risk increases each year as they age. Multiplying that mortality risk over their future life forms the basis of their estimated life expectancy. It is hard to imagine what a death rate or risk means to people: the life course perspective has meaning to them since they can imagine what it means to live to 80, 85 or 90 years. Similar methodologies are used for calculating other health risks, such as risk of having a heart attack or stroke.

Table 1 shows a list of online life expectancy and health risk calculators linked to the 2020 Canadian Health Clock. These calculators were developed using the CCHS linked to mortality and disease incidence, and are published in peer-reviewed journals (Manuel et al. 2016, 2018a, b; Arcand et al. 2014; Wang et al. 2013). These calculations used population-based self-reported health risks, as opposed to clinical measures that are more commonly used in clinical risk algorithms. The large size of the CCHS allows statistical power for a “precision health” approach whereby risk is discriminately calculated for a person’s individual health profile. The CCHS has some limitations, such as not including First Nations and some other specific populations, but it is the main population health surveillance survey for important measures such as socio-demographic (e.g., education, immigration, income) and health behaviours (smoking, alcohol, diet and physical activity). As such, these linked data are well suited for the development of health calculators with a public health focus.

**Table 1:** Online life expectancy and health calculators that are linked to the 2020 Canadian Health Clock

Calculators	Description
(1) Life Expectancy Calculator (Manuel et al. 2016; Manuel et al. 2018a) https://www.projectbiglife.ca/life-expectancy-home	How long can I expect to live? This calculator also suggests ways to live a longer life.
(2) Heart Attack and Stroke Calculator (Manuel et al. 2018b) https://www.projectbiglife.ca/cardiovascular-disease	What’s my risk of having a heart attack or stroke? This calculator predicts the risk of developing cardiovascular disease over the next 5 years.
(3) Sodium Calculator (Arcand et al. 2014) https://archive.projectbiglife.ca/sodium/	How much salt do I eat in a day? This calculator shows how personal daily sodium intake can be reduced.
(4) Predicting Depression Calculator (Wang et al. 2013) http://predictingdepression.com/	Predicting future risk of having major depression. This calculator predicts the risk of developing major depression over the next 4 years.

## Preliminary evaluation of the clock and calculators

Health Portfolio staff provided feedback (*N* = 29 respondents) in a preliminary evaluation survey of the clock and calculators conducted in June 2019 (Table 2). First, most respondents thought the clock has a very positive (34.5%) or a somewhat positive (34.5%) impact on public health practice or policy in Canada. On the other hand, some thought the clock has a somewhat negative impact (6.9%). Second, most thought the clock is somewhat innovative (55.2%). On the other hand, some thought the clock is not at all innovative (6.9%). Third, many thought the online calculators will somewhat likely (41.4%) help move people toward behaviour change. On the other hand, many thought this will be very unlikely (24.1%). Fourth, most thought journal publications would very likely (55.2%) or somewhat likely (27.6%) endorse the scientific value and promote the use of the calculators. Typical comments representing a wide range of opinions are provided in Table 2.

**Table 2:** Results of a preliminary evaluation of the Canadian Health Clock and health calculators from a survey of Health Portfolio staff conducted in June 2019 (*N* = 29)

	a. Very positive/very innovative/very likely	b. Somewhat positive/somewhat innovative/somewhat likely	c. Neutral	d. Somewhat negative/not so innovative/somewhat unlikely	e. Very negative/not at all innovative/very unlikely
1. Do you think the Canadian Health Clock has a positive or negative impact on public health practice or policy in Canada?	10 (34.5%)	10 (34.5%)	7 (24.1%)	2 (6.9%)	0 (0.0%)
2. How innovative is the Canadian Health Clock?	6 (20.7%)	16 (55.2%)	4 (13.8%)	1 (3.4%)	2 (6.9%)
3. What makes the Canadian Health Clock innovative to public health practice or policy in Canada?	*Comments under (A) in footnote below	*Comments under (B)	*Comments under (C)	*Comments under (D)	*Comments under (E)
4. Do you think the online calculators provided on the Canadian Health Clock website will likely help move people toward behaviour change?	3 (10.3%)	12 (41.4%)	4 (13.8%)	3 (10.3%)	7 (24.1%)
5. Online health calculators are now quite widespread. All of the calculators being promoted on the Canadian Health Clock website have been published in peer-reviewed journals. Would journal publication be your consideration that these calculators are better than their many competitors available online, that they are based on stronger incorporation of science, and that the public should trust these calculators more than others?	16 (55.2%)	8 (27.6%)	4 (13.8%)	0 (0.0%)	1 (3.4%)

*Comments for Question 3:

(A) Very innovative: “Visual representations of data are significantly more impactful than numerical data.”; “The clock brings vital statistics to life and imbues them with a sense of urgency.”; “This is fascinating. It really brings home the point that many people are dying every day of various diseases.”; “It is interesting to see the data accumulate in real time.”; “Moving circles that replace second hands on a clock make it easier to visualize an approaching death when a circle is complete.”

(B) Somewhat innovative: “I will not call it as an innovation. However, I do believe that the clock has much more impact than data presented in a graph or tabulated form. The number may not be accurate but gives a sense on how big the issue is. It is assumed that the clock will draw attention of the general public and also the decision makers to act accordingly.”; “There are other health clocks out there, but I believe this is the only one using Canadian data, is probably the most opened in terms of methodology used, and connected to validated and scientifically sound health risk calculators.”; “I like the look of the clock and the interaction one can have with it. But the clock needs to be linked to sites to inform one to make healthy life choices (food, exercise etc.…)”; “A health clock is not a new idea, but I do not ever recall it being done previously within the Public Health Agency of Canada, so I’m impressed with the tool and will definitely share with family and friends once it’s officially released.”

(C) Neutral: “The data contained in the clock may be helpful for public health priority setting/practice and policy, if the leading causes of death information presented was also available by age and sex as the profile of the rankings will change with those breakdowns and allow public health to focus specifically at those at most risk, e.g. youth suicide.”; “Data visualizations are key these days, but I’m not sure the emphasis on deaths is the right approach.”

(D) Not so innovative: “Which, of course, presumes that the clock is deemed innovative. Talk about a leading question …”

(E) Not at all innovative: “It is not innovative. It is simplistic and is purely an estimation of daily number of deaths based on total number of death from retrospective data that have been divided by 365 days and then 1440 minutes. This ignores all sorts of issues, such as seasonality, which affects death distributions and the fact that everyone dies (it is all deaths, instead of premature deaths that would give you some actual interesting information).”; “I do not think that the way the graphic displays the count of deaths is intuitive to people.”

The role of personal risk communication for the mortality risk calculator was examined in another survey of 317 respondents (Manuel et al. 2018c). Over 80% of respondents found at least one personalized mortality measure “highly” informative and motivating. Within all respondents, health age was most informative, whereas life expectancy was most motivating. There was further heterogeneity across socio-demographic groups, suggesting a role for multiple measures for presenting risk within personalized reports.

It is a common misconception that health risk assessment, by itself, is a behaviour change intervention. Rather, the purpose of health risk assessment is a health communication tool or a health risk stratification tool for identifying people who may benefit from interventions. Calculating personal risk identifies and characterizes potential intervention benefit, but should be coupled with health behaviour modification or other health interventions (Michie et al. 2011). While not extensively studied as a health intervention, a Cochrane review of cardiovascular risk assessment showed “providing cardiovascular risk scores may slightly reduce cardiovascular risk factor levels and may increase preventive medication prescribing in higher-risk people without evidence of harm” (Karmali et al. 2017a).

## Epilogue

Both the clock concept and the various health calculators are not new, and are now quite widespread on the Internet. A popular clock is the World Health Clock (World Clock n.d.). It has deaths this year and today for 19 causes of death in the world and major regions (such as “Western Europe and North America”). Its Canada clock, however, has only deaths for 6 seemingly randomly chosen causes (cancer, heart disease, stroke, emphysema and bronchitis, motor vehicle fatalities, and suicide). Furthermore, this clock webpage describes neither the methodology nor the data source. Another website describes 135 health calculators that are sorted alphabetically (MedIndia n.d.). The calculators, however, have not been peer reviewed in scientific publications. Many are overly simplistic; for example, its life expectancy calculator is based on only two variables (your age and your country).

There are several advantages that make the versions described in this paper innovative to public health practice and policy in Canada. As concisely summarized in a comment in our preliminary evaluation survey, “There are other health clocks out there, but I believe this is the only one using Canadian data, is probably the most opened in terms of methodology used, and connected to validated and scientifically sound health risk calculators”. Another innovation in this project is to link up the clock and the calculators, something which has not been attempted in any other websites. We believe the calculators being promoted in this paper are better than the many competitors available online because of a stronger incorporation of science and having been published in peer-reviewed journals. For example, our life expectancy calculator is based on a careful review of the scientific literature, follows a robust computational algorithm and is based on multivariable personal inputs including both non-modifiable risk factors such as age, sex and ethnicity, and modifiable risk factors such as smoking, physical activity and nutrition (Manuel et al. 2016, 2018a). In general, the public should trust clocks and calculators which have a clear accountability of the methodology and data source, and which have been peer reviewed, more than others.

The Canadian Health Clock is a counter of deaths. We chose the name health clock because it has a more positive connotation than a death clock, and because a death clock is defined in the dictionary as “a clock that displays when a person will die” (Your Dictionary n.d.), which is not our clock. Another dictionary gives two definitions: “(1) A metaphorical clock representing when one is expected to die; (2) (*humorous*) Any of various websites that attempt to calculate when a person will die” (Wiktionary n.d.). Our clock is a good example of innovation in effective knowledge translation and dissemination. Instead of telling people the number of deaths in a year, which is an enormous number difficult to comprehend, the clock shows the number of deaths since the beginning of the year, and since midnight last night, which are smaller numbers to which people can relate. The 2020 Canadian Health Clock has pushed data visualization to the next level, with moving circles, blinking red dots and growing bar charts. There are more than a hundred life expectancy calculators found online, but many of them are non-scientific and therefore more for fun and humorous purposes; for example, by telling people their exact date of death and exact days left to live.

Informed healthy public policy implies that people know how policy and health risks affect their community (the clock) and themselves (personal risk calculators). The public health objective of health promotion tools is to inform and engage people in policy to improve communication and engage people in health information (Milkovich et al. 2013). By itself, there is limited evidence that health information including the clock and personal health calculators affect behaviour change (Karmali et al. 2017b). From a public health perspective, however, the lack of behaviour change is unsurprising without supportive environments and specific health interventions (Michie et al. 2011). Similarly, the science of health communication is evolving, with more research needed to assess how different measures are cognitively interpreted and their role in behaviour change (Bennett et al. 2010). For example, there is good evidence that iconic representation of deaths improves understanding, but clocks have not been extensively evaluated (Ancker et al. 2006). That stated, there is increasing evidence to suggest personal risk assessment as an important component of multi-component personalized health interventions. Personal risk estimation shifts public health recommendations from one-size-fits-all recommendations to preventive recommendations that are evidence-informed and value-congruent to individuals, rather than to populations.

Following the celebration activities in Canada of “100 Years of Health”, it is timely to announce the release of the 2020 Canadian Health Clock which is the product of one celebration activity, and a linkage to a repertoire of scientific online calculators, as part of the ongoing efforts for improving health for all.
